# Supplementary material for: Sorting at embryonic boundaries requires high heterotypic interfacial tension
Source: Nat Commun. 2017 Jul 31;8:157. doi: 10.1038/s41467-017-00146-x (PMC5537356; doi:10.1038/s41467-017-00146-x)
Supplement: Supplementary file 2 — Supplementary Software 1 [file 41467_2017_146_MOESM2_ESM.zip › PottsModel/SrcPottsModel/doc/index-files/index-4.html]

D-Index


JavaScript is disabled on your browser.


Skip navigation links


- Overview
- Package
- Class
- Use
- Tree
- Deprecated
- Index
- Help

- Prev Letter
- Next Letter

- Frames
- No Frames

- All Classes

A B C D E F G H I J K L M N O P R S T U V W 


## D

decreaseFactorAdhesion - Variable in class model.Constants.ParameterManager


decreaseFactorTemperature - Variable in class model.Constants.ParameterManager


decreaseTime - Variable in class model.Constants.ParameterManager


DEFAULT\_FREQUENCY - Static variable in class engine.Statistic


DefaultSimulation - Class in simulations


DefaultSimulation() - Constructor for class simulations.DefaultSimulation


disableLogger(Logger) - Static method in class utils.IO


disableSaving() - Method in class model.Constants


DispersionIndex - Class in engine
:   Dispersion index statistic measured for each cell type using 10x10 quadrats.

DispersionIndex(PottsEngine, Cell.CellType[], int) - Constructor for class engine.DispersionIndex


DispersionIndex(PottsEngine, Cell.CellType[]) - Constructor for class engine.DispersionIndex


displayCellInformation(int, int) - Method in class gui.PottsFrame


displayPixelInformation(int, int) - Method in class gui.PottsFrame


distance(Hexagon.CubeCoordinates, Hexagon.CubeCoordinates) - Static method in class gui.Hexagon


distance(Hexagon.AxialCoordinates, Hexagon.AxialCoordinates) - Static method in class gui.Hexagon


distance(Hexagon.OffsetCoordinates, Hexagon.OffsetCoordinates) - Static method in class gui.Hexagon


DITHSimulation - Class in simulations
:   A simple simulations using the common-properties configuration.

DITHSimulation() - Constructor for class simulations.DITHSimulation


doVectorTransform(Point) - Method in class model.FiniteVector


draw(Graphics2D) - Method in class gui.CellDisplay


drawColor(Graphics2D) - Method in class gui.PixelDisplay


drawEdge(Graphics2D, PixelShape.Edge, boolean) - Method in class gui.PixelDisplay
:   Set edge as active/inactive and then draw it.

drawEdges(Graphics2D) - Method in class gui.PixelDisplay

A B C D E F G H I J K L M N O P R S T U V W

Skip navigation links


- Overview
- Package
- Class
- Use
- Tree
- Deprecated
- Index
- Help

- Prev Letter
- Next Letter

- Frames
- No Frames

- All Classes
